# Epitranscriptomic profiling of cytosine N4 acetylation (ac^4^C) in *Solanum lycopersicum* and dynamic changes under heat stress condition

**DOI:** 10.1186/s43897-025-00214-7

**Published:** 2026-05-06

**Authors:** Wanhong Zhang, Yubing Jiao, Yanxiao Bu, Lili Shen, Yingwen Wang, Ying Li, Binna Lv, Qi Huang, Yansong Xiao, Tianbo Liu, Jinguang Yang

**Affiliations:** 1 Key Laboratory of Tobacco Pest Monitoring Controlling & Integrated Management, Tobacco Research Institute of Chinese Academy of Agricultural Sciences, Qingdao, 266100 China; 2 China Tobacco Hunan Industrial Co., Ltd, Changsha, China

**Keywords:** *S. lycopersicum*, ac^4^C, Heat stress, acRIP-seq, RNA stability

## Abstract

N4-acetylcytidine (ac^4^C), which is an evolutionarily conserved RNA modification in eukaryotes, functions as a critical epitranscriptomic regulator that enhances mRNA stability and translation efficiency. In *Arabidopsis thaliana* and *Oryza sativa*, ac^4^C has been characterized as a modulator of transcriptome homeostasis that functions by regulating translational efficiency and maintaining RNA structure; however, the distribution patterns and biological functions of ac^4^C in other plant species remain largely unexplored. A transcriptome-wide map of the ac^4^C of *Solanum lycopersicum* revealed conserved features of plant ac^4^C modification, with preferential enrichment in mRNA translation initiation and termination regions as well as high conservation of modification motifs and functional categories of target genes across species. Notably, heat stress triggered global ac^4^C hyperacetylation, and hypermodified genes were enriched in transcripts encoding photosynthesis and thermotolerance-associated regulators. Multiomics integration further revealed that hyperacetylated and upregulated genes were significantly enriched in stress-response pathways, accompanied by elevated transcription levels. Silencing of SLNAT10 resulted in reduced stability of highly acetylated transcripts under heat stress conditions. These findings reveal that RNA acetylation is synchronized with posttranscriptional regulation that promotes transcriptional reprogramming to establish heat tolerance, providing a mechanism for the epitranscriptomic control of environmental adaptation in plants.

**Supplementary Information:**

The online version contains supplementary material available at 10.1186/s43897-025-00214-7.

## Core

Under heat stress conditions, a global increase in ac^4^C acetylation is observed in *S. lycopersicum*. Hyperacetylated genes are significantly enriched in pathways related to photosynthesis and thermotolerance, and exhibit coordinated transcriptional upregulation as well as enhanced posttranscriptional stability.

## Gene & accession numbers 

Information on the genes in this study can be found in the database (Sol Genomics Network: https://solgenomics.net) under the accession number SLNAT10 (Solyc04g051670.2.1)

## Introduction

In epitranscriptomics, more than 170 distinct types of RNA chemical modifications have been identified, highlighting the roles of these modifications as post-transcriptional regulatory elements (Zhao et al. 2017). These modifications are critical for carefully regulating gene expression, maintaining RNA stability, and ensuring accurate translation; thus, their discovery has expanded our understanding of the complex regulatory network governing RNA metabolism (Nachtergaele and He 2018). Chemical modifications have been detected in nearly all types of cellular RNA, including both coding RNAs and noncoding RNAs, such as tRNAs, rRNAs, lncRNAs, and miRNAs (Roundtree et al. 2017). Among these modifications, methylation is the most extensively studied category, with prominent examples including N⁶-methyladenosine (m⁶A), 5-methylcytosine (m^5^C), N⁷-methylguanosine (m⁷G), pseudouridine (Ψ), and N^1^-methyladenosine (m^1^A). In contrast, research on RNA acetylation modifications remains limited to a few known forms, with ac^4^C being the most representative type. This modification primarily occurs on the amino group of cytidines, resulting in the formation of N^4^-acetylcytidine. The first ac^4^C modification was discovered in the tRNA-Met of *Saccharomyces cerevisiae* (Oashi et al. 1972), and subsequent studies revealed its presence in mRNA and rRNA across eukaryotic systems, including humans and yeast, confirming its conservation across species (Thomas et al. 2018). The enzymatic deposition of ac^4^C is catalyzed by ATP-dependent acetyltransferases (Ito et al., 2014), in which human NAT10 and yeast Kre33 serve as the primary “writer” proteins (Sharma et al. 2015). These enzymes utilize acetyl-CoA as the acetyl donor to complete the modification.

ac^4^C is distinct from the extensively studied m⁶A modification, and it is recognized for its role in maintaining RNA stability by enhancing stability and translational efficiency across various biological contexts (Arango et al. 2018). In mRNA molecules, ac^4^C modification improves stability and translational accuracy by modulating their secondary structures (Dominissini et al. 2018). In tRNAs, ac^4^C is concentrated in the anticodon loop, where it optimizes folding and the decoding efficiency to ensure accurate protein synthesis. Within ribosomal RNA (rRNA), ac^4^C is essential for the proper assembly of ribosomal subunits; the absence of this modification is associated with reduced protein synthesis efficiency, metabolic disruption and impaired cellular function. Moreover, ac^4^C performs significant functions under various physiological and pathological conditions. NAT10 expression is upregulated following DNA damage, enhancing cellular resistance to H₂O₂-induced senescence (Liu et al. 2016). In breast cancer, NAT10 overexpression increases the translational efficiency of tumor-associated proteins such as EGFR, thereby driving tumor progression (Wei et al. 2023). Additionally, Human Immunodeficiency Virus (HIV) exploits host-derived NAT10 to modify vRNA, thus enhancing viral translation and proliferation (Tsai et al. 2020). These findings highlight that ac^4^C modifications not only regulate RNA stability and functional regulation but also participate in stress responses, gene regulation, and pathological processes through diverse molecular mechanisms, establishing their potential for use as therapeutic targets and biomarkers.

In plants, research on ac^4^C modification is in rapidly emerging research. acRIP-seq has mapped the distribution of ac^4^C in *Arabidopsis thaliana* and *Oryza sativa*, revealing its contributions to RNA stability and splicing regulation (Li et al. 2023). This modification regulates mRNA translational reprogramming and plays a crucial role in plant immune responses. The acetyltransferase OsNAT10/OsACYR enhances immune-related gene translation and activates jasmonic acid biosynthesis during immune activation (Lu et al. 2024). Futhermore, the deep learning model “iac^4^C” has been used to identify specific patterns of ac^4^C modification in plant RNA transcriptional regions, highlighting its role in alternative splicing regulation (Guo et al. 2024). In tomato fruit, ac^4^C modifications are enriched in fruit ripening-related genes and influence ethylene production, fruit texture, and flavor (Ma et al. 2024). Building on these findings, acRIP-seq was performed in this study to detect and map transcriptome-wide ac^4^C patterns in tomato plants and to analyze the distribution patterns of ac^4^C modifications in mRNA from tomato leaves. Through the combined analysis of acRIP-seq and RNA-seq, the dynamic changes in mRNA acetylation modifications in tomato plants under heat stress conditions and their impact on mRNA transcription levels were investigated. Our results reveal potential regulatory mechanisms of ac^4^C in response to environmental stress and its impact on mRNA stability.

## Results

### Presence of cytosine N4 acetylation (ac^4^C) in *S. lycopersicum* mRNAs

**Fig. 1:**
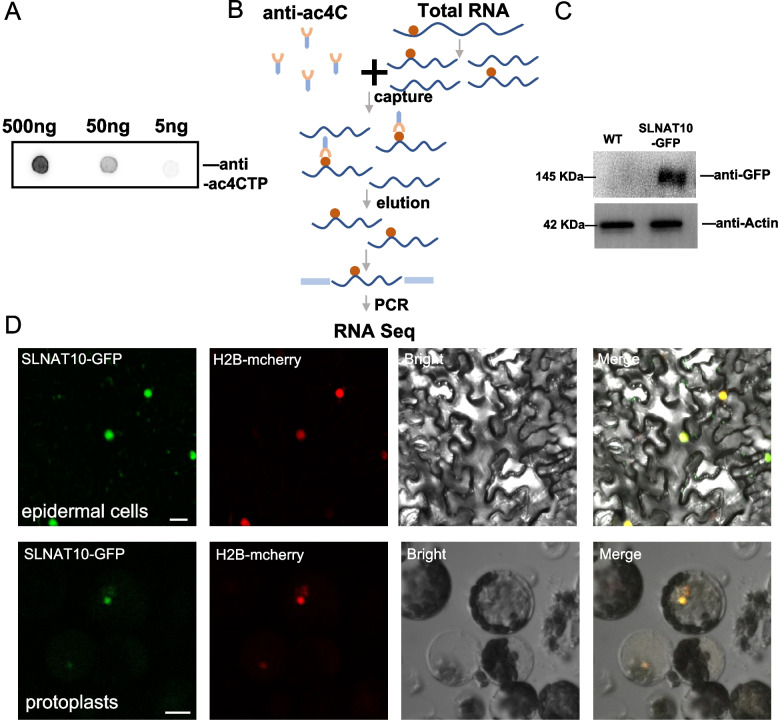
Detection of ac^4^C in tomato mRNA and the subcellular localization of SLNAT10. **A** RNA dot blotting analysis of ac^4^C modification in tomato mRNA. Serial tenfold dilutions (500 ng to 5 ng) were probed with an anti-ac^4^C antibody, which showed robust ac^4^C-specific signals. **B** Schematic workflow of acRIP-seq to identify ac^4^C-modified mRNAs in *S. lycopersicum.* **C** Western blotting analysis confirmed the expression of the SLNAT10-GFP fusion protein. **D** Nuclear localization of SLNAT10-GFP in tomato leaf epidermal cells and protoplasts. Colocalization with the nuclear marker H2B-mCherry confirms exclusive nuclear targeting. Scale bar = 20 μm

RNA ac^4^C modification has been validated in the mRNAs of *A. thaliana* and *O. sativa* (Li et al. 2023; Wang et al. 2023). This modification in mRNAs is highly abundant and thought to regulate gene expression or maintain mRNA stability. To determine whether ac^4^C modification occurs in tomato plants, total RNA was extracted from the leaves of 6-week-old tomato plants, and mRNA was subsequently purified using oligo dT-coated magnetic beads. The presence of ac^4^C modifications in tomato mRNA was initially evaluated by RNA dot blotting analysis (Fig. 1A). Significant ac^4^C-specific signals were observed. To map modification sites genome-wide, acRIP-seq was performed. RNA fragments containing ac^4^C modifications were immunoprecipitated with an ac^4^C-specific antibody (Fig. 1B), high-quality libraries were sequenced, and the clean reads were aligned to the tomato reference genome (Sol Genomics Network: https://solgenomics.net) to identify ac^4^C-modified transcripts.

To identify the acetyltransferase responsible for ac^4^C deposition, a BLAST search against publicly available NAT10 sequences from *A. thaliana* and *O. sativa* revealed a single-copy homolog in tomato, designated SLNAT10 (Solyc04g051670.2.1). Sequence alignment revealed that the amino acid sequence of tomato RNA cytidine acetyltransferase is similarity with yeast Kre33, human NAT10, and *A. thaliana* NAT10 (Figure S1A). Further phylogenetic analysis with other plant species revealed that SLNAT10 is similar to NAT10 in plants from distinct families but is not clustered on the same branch as NAT10 homologs from other *Solanaceae* species are (Figure S1B). Subcellular localization analysis revealed that EGFP-tagged SLNAT10 exclusively localized to the nucleus in both tomato leaf epidermal cells and protoplasts, which is consistent with the nuclear function of NAT10 orthologs in other species. Moreover, western blotting analysis confirmed the expression of SLNAT10 in tomato leaves (Fig. 1C and Figure S2).

### Analysis of *S. lycopersicum* RNA ac^4^C acetylomes

**Fig. 2:**
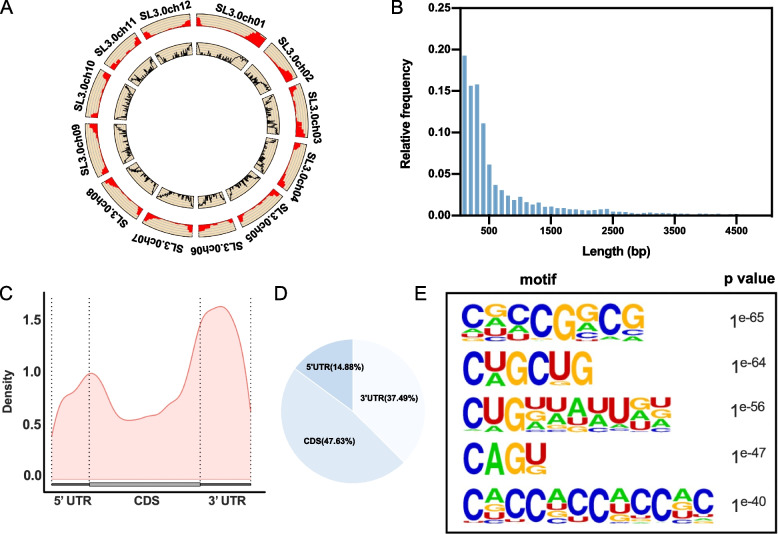
Genome-wide distribution and sequence motifs of ac^4^C peaks in tomato. **A** Circos plot visualizing ac^4^C modification patterns across the tomato genome. Two rings from outside to inside show the distribution density of ac^4^C modification on the genome (1st) and the degree of enrichment of ac^4^C modification on chromosome segments (2nd). **B** Length distribution of ac^4^C peaks in target genes. **C** Metaplot showing the distribution of ac^4^C peaks across tomato transcripts. The relative positions of the UTRs and CDS are shown at the bottom. **D** Proportional distribution of ac^4^C-modified transcripts across different functional regions of genes. **E** The top five conserved motifs identified in the ac^4^C peaks

The acRIP-seq experiments were performed in three biological replicates, with each sample yielding a minimum data volume of 7.48 Gb and clean reads Q30 scores exceeding 90%. High-quality sequencing reads were aligned to the tomato reference genome using STAR software, generating 7–20 million reads mapped to the tomato genome. Each sample demonstrated a mapping rate above 90%, confirming the precision of acRIP-seq in detecting ac^4^C modifications within tomato mRNAs (Supplementary material 2). Genome-wide analysis revealed 6,311 ac^4^C peaks localized to 5,167 genes, with an even distribution across all 12 tomato chromosomes (Fig. 2A). Peak length analysis revealed a predominant size range of 75–500 bp (Fig. 2B). Gene annotations indicated that ac^4^C modification was enriched near the start codon (Fig. 2C), mirroring patterns observed in human, *A. thaliana*, and *O. sativa* mRNAs (Dominissini et al. 2018; Li et al. 2023; Wang et al. 2023). Notably, unlike those in *A. thaliana* and *O. sativa*, 31.3% of the identified ac^4^C modifications were concentrated in the 3' untranslated region (UTR), whereas nearly 47.47% were located in the noncoding regions of genes (Fig. 2D). To identify conserved ac^4^C motifs, HOMER software was used to analyze shared peaks, extract sequence intervals, and visualize enriched motifs. Two enriched motifs, CNHCGVCV and CWGCKG, were identified (Fig. 2E). Although lacking the strong periodicity seen in *A. thaliana* and *O. sativa* (CUUCUUCYUCYU ranks high in *A. thaliana* and CNNCNNCNNC ranks high in *O. sativa* (Li et al. 2023)), they follow a CNN-like backbone and show C enrichment with CG-centered cores, indicating partial conservation at the compositional level.

### Enrichment analysis of ac^4^C-enriched RNA in tomato

**Fig. 3:**
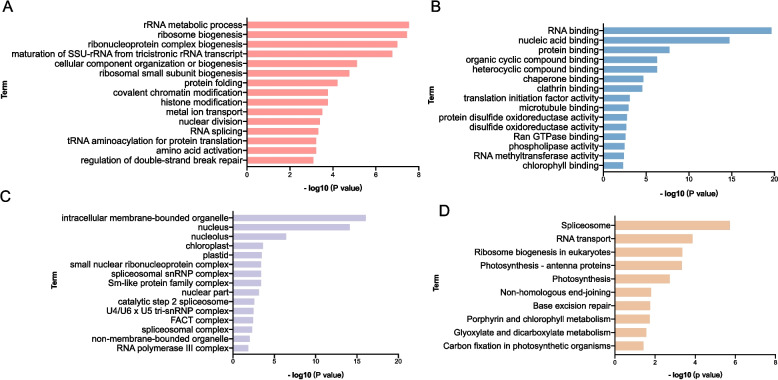
Enrichment analysis of ac^4^C-modified transcripts in tomato. **A** GO enrichment analysis of biological processes for tomato ac^4^C-modified transcripts. **B** GO enrichment analysis of molecular functions for tomato ac^4^C-modified transcripts. **C** GO enrichment analysis of cellular components for tomato ac^4^C-modified transcripts. **D** KEGG enrichment analysis for tomato ac^4^C-modified transcripts

To elucidate the potential biological functions of ac^4^C modification in tomato, genes with ac^4^C modification peaks were subjected to Gene Ontology (GO) and KEGG pathway analyses. GO enrichment analysis of biological processes suggested that ac^4^C-modified RNAs are associated with critical processes, including rRNA metabolic processes, ribosome biogenesis, and mRNA splicing, suggesting their central role in regulating gene expression and protein synthesis. Molecular function analysis revealed enrichment in RNA binding, nucleic acid binding, and protein binding, directly linking ac^4^C to RNA–protein interactions and posttranscriptional regulation. Cellular component analysis further localized these modifications to functional hubs of RNA processing, including the nucleus, nucleolus, and ribosomes, with additional enrichment in mitochondria and chloroplasts, suggesting potential roles in energy metabolism and photosynthesis. KEGG pathway analysis revealed that ac^4^C-modified RNAs were enriched in the terms “spliceosome”, “RNA transport”, and “ribosome biogenesis”, highlighting their importance in RNA maturation and translation. Notably, photosynthesis-related pathways highlight potential connections to chloroplast gene expression and light-dependent processes. Collectively, these findings establish ac^4^C as a multifaceted regulator that orchestrates RNA metabolism, translational fidelity and organelle functions in tomato plants (Fig. 3).

### Profile of ac^4^C acetylation in heat-stressed tomato

**Fig. 4:**
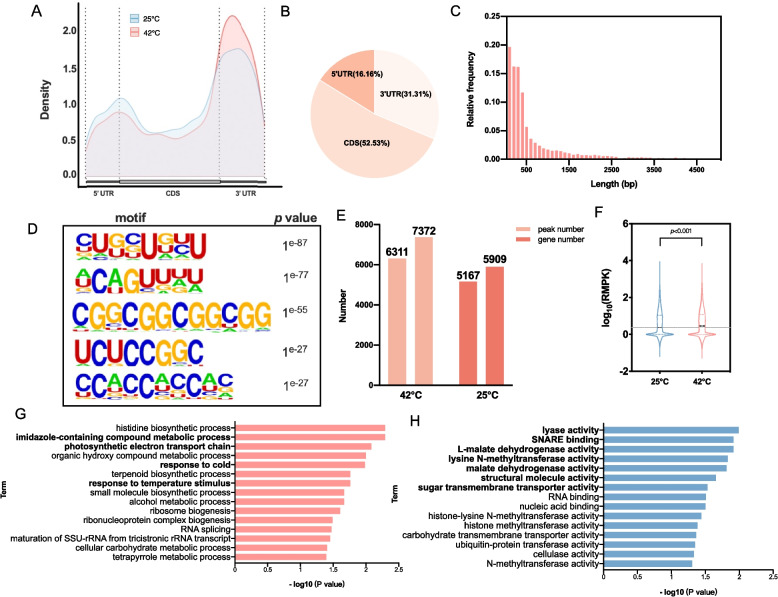
Heat stress-induced dynamics of ac^4^C modifications in tomato transcripts. **A** Metaplot showing the distribution of ac^4^C peaks under control and heat stress condition. The relative positions of the UTRs and CDS are shown at the bottom. **B** Pie chart displaying the proportional distribution of ac^4^C peaks among different transcript regions (5'UTR, CDS, 3'UTR) in tomato genes under heat stress condition. **C** Histogram showing the length distribution of genes associated with ac^4^C peaks across tomato transcripts under heat stress condition. **D** The top five sequence motifs identified from ac^4^C-enriched transcripts under heat stress condition. **E** The number of ac^4^C peaks and associated genes across tomato transcripts under normal growth conditions (25 °C) and heat stress condition (42 °C). **F** Boxplot of RNA modification levels (log_10_(RPKM)) in tomato plants subjected to 25 °C or 42 °C conditions. RNA modification levels were significantly higher at 42 °C than at 25 °C (p < 0.001, Welch’s t-test). **G** GO enrichment analysis of the biological processes of the DMGs. **H** KEGG enrichment analysis of the DMGs

The conserved role of ac^4^C in thermal adaptation is supported by its ability to stabilize RNA under heat stress in *Thermococcus kodakarensis* (Sas-Chen et al. 2020), suggesting evolutionary conservation of this modification in stress responses. To explore its functional relevance in plants, acRIP-seq analysis was performed on mRNAs from heat-stressed tomato plants. The results indicated that heat stress did not cause notable changes in the distribution of ac^4^C modifications across gene functional regions (Fig. 4A-B) or the peak lengths of ac^4^C modifications (Fig. 4C). Notably, however, the sequence motifs associated with the ac^4^C peaks exhibited distinct patterns under heat stress conditions: the highest-ranking motif was identified as CWG, but most of the top-ranked motifs retained the characteristic CNN sequence pattern (Fig. 4D). Quantitative analysis revealed an increase in both the number of ac^4^C peaks and the associated genes, as the peak count increased from 5,167 to 6,311 and the number of genes containing these peaks increased from 7,372 to 8,824 (Fig. 4E). Furthermore, the level of the ac^4^C modification levels significantly increased under heat stress conditions (Fig. 4F).

DESeq2 was used to identify genes that were significantly modified in tomato plants under heat stress conditions compared with room-temperature conditions, and 2,721 differentially modified genes (DMGs) were identified. GO enrichment analysis of the DMGs revealed significant alterations in molecular functions and biological processes. Compared with those under non-heat-treated conditions, the results highlighted several stress-responsive pathways, with a particular focus on activity-related molecular functions and heat-adaptive biological processes. Biological processes were dominated by heat-responsive pathways, including “response to heat” and “response to temperature stimulus” (Fig. 4G), directly linking ac^4^C to transcriptional reprogramming during thermal adaptation. Additionally, enrichment of the photosynthetic electron transport chain suggests that ac^4^C-mediated regulation is potentially needed to preserve photosynthetic efficiency under stress conditions. Metabolic pathways were also significantly enriched, indicating an adaptive shift toward stress-related secondary metabolite production. Molecular function analysis highlighted activity-related terms and demonstrated the importance of ac^4^C modifications in enzymatic and protein regulation during heat stress. Enrichment of “lyase activity” and “malate dehydrogenase activity” indicated the role of ac^4^C in redirecting carbon metabolism for energy homeostasis, whereas “SNARE binding” enrichment indicated enhanced vesicle trafficking for cellular logistics under stress (Fig. 4G). Collectively, these findings suggest that ac^4^C contributes to tomato heat tolerance by influencing transcriptional reprogramming, metabolic adaptation, and cellular protection through RNA modification.

### Integrative analysis of transcriptomic and ac^4^C epitranscriptomic changes in tomato under heat stress condition

**Fig. 5:**
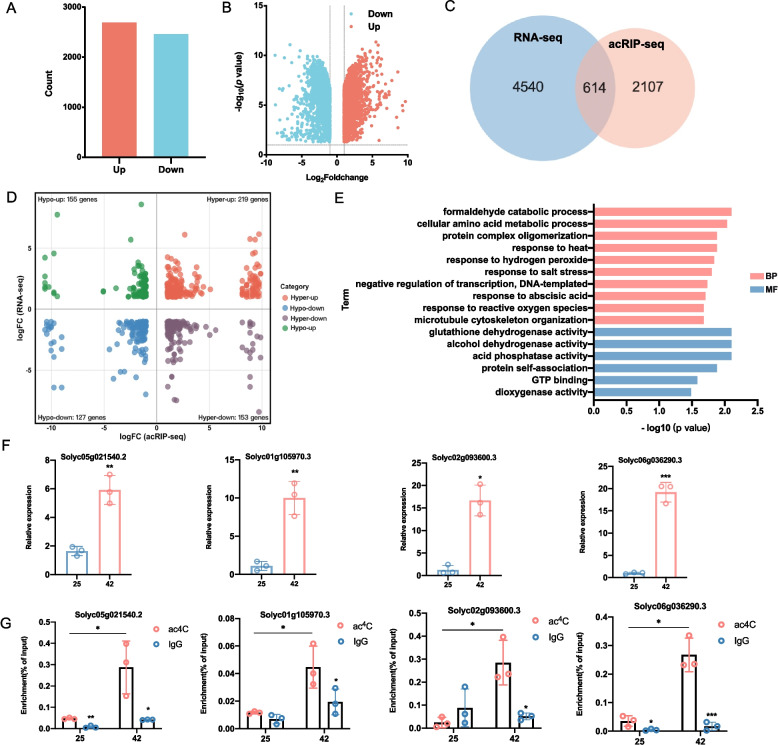
Integrated analysis of transcriptional and ac^4^C modification dynamics under heat stress. **A** A bar chart showing 5,154 differentially expressed genes (DEGs) at the transcriptional level following HS treatment, including 2,694 upregulated and 2,460 downregulated genes. **B** Volcano plot showing the distribution of DEGs, including 2,694 upregulated (red) and 2,460 downregulated genes (blue). **C** A Venn diagram illustrating the overlap of 614 genes whose DMGs and DEGs are significantly altered. **D** A quadrant plot visualizing changes in transcription and acetylation levels for the 614 common genes, differentiating the hyper-up, hyper-down, hypo-up and hypo-down groups. **E** Gene Ontology (GO) enrichment analysis was performed on the hyper-up group of 219 genes. **F** RT‑qPCR results show significant upregulation of the transcription levels of the four candidate genes under heat stress condition. The data represent the mean ± SD of three biological replicates. Statistical significance was determined using one-way ANOVA (*p < 0.05, **p < 0.01, ***p < 0.001). **G** acRIP-RT-qPCR results show a corresponding increase in ac^4^C modifications for the same genes. The data represent the mean ± SD of three biological replicates. Statistical significance was determined using one-way ANOVA (*p < 0.05, **p < 0.01).

To investigate the impact of ac^4^C modification on mRNA transcription under heat stress condition, RNA-seq analysis was employed to profile transcriptional changes in tomato mRNAs exposed to elevated temperatures. The analysis revealed 5,154 differentially expressed genes (DEGs), comprising 2, 694 upregulated genes and 2, 460 downregulated genes, following heat treatment (Fig. 5A-B). A combined analysis was performed by comparing genes with significant transcriptional changes to those with significant ac^4^C modification alterations. Integration with the ac^4^C modification data revealed 614 overlapping genes (Fig. 5C), which were classified into four groups on the basis of log_2_(fold-change) values of transcription and acetylation levels (Fig. 5D). Among the four categories, the hyper-up group, consisting of 219 genes, was notably larger than the other three categories, indicating a positive correlation between ac^4^C modifications and the upregulation of transcription. These findings suggest that ac^4^C modifications potentially increase transcriptional activity under stress conditions.

GO analysis of the hyper-up group (Fig. 5E) highlighted stress-responsive biological processes such as “formaldehyde catabolic process”, “response to reactive oxygen species (ROS)”, “response to heat”, and “response to hydrogen peroxide”, indicating their role in mitigating heat-induced cellular damage. The molecular function category featured enzymatic activities (glutathione dehydrogenase, alcohol dehydrogenase, acid phosphatase, and dioxygenase), linking ac^4^C to metabolic rewiring and detoxification. These findings collectively suggest that ac^4^C plays a role in coordinating transcriptional resilience under heat stress. Experimental validation (Fig. 5F, G) targeted four hyper-up genes (Solyc06g036290.3, Solyc05g021540.2, Solyc01g105970.3, and Solyc02g093600.3) in 6-week-old tomato plants subjected to 42 °C (12 h) versus 25 °C controls. The levels of ac^4^C modification and transcription expression of the selected genes were analyzed under high-temperature and control conditions. After verifying RNA integrity by agarose gel electrophoresis for six samples (Figure S3), the results of acRIP-RT-qPCR demonstrated a significant increase in ac^4^C modifications for the four candidate genes under heat stress condition, while RT‑qPCR analysis revealed a concomitant significant upregulation of their transcription levels (Fig. 5F-G). RT‑qPCR analysis revealed transcriptional upregulation of the tested genes under heat stress condition (Fig. 5F). Strikingly, acRIP-RT-qPCR demonstrated a parallel increase in the ac^4^C modification levels of these genes (Fig. 5G). Meanwhile, the validation results for two hyper-down genes (Solyc10g050460.1, Solyc01g091320.3) by RT-qPCR and acRIP-RT-qPCR showed that the transcription levels of these two candidate genes are significantly reduced under heat stress (Figure S4A-B), and a corresponding decrease in ac^4^C modifications for the same genes (Figure S4C-D). This coordinated in transcription and acetylation is consistent with a mechanistic model in which ac^4^C modifications may enhance mRNA stability, thereby contributing to amplified stress‑responsive gene expression.

**Fig. 6:**
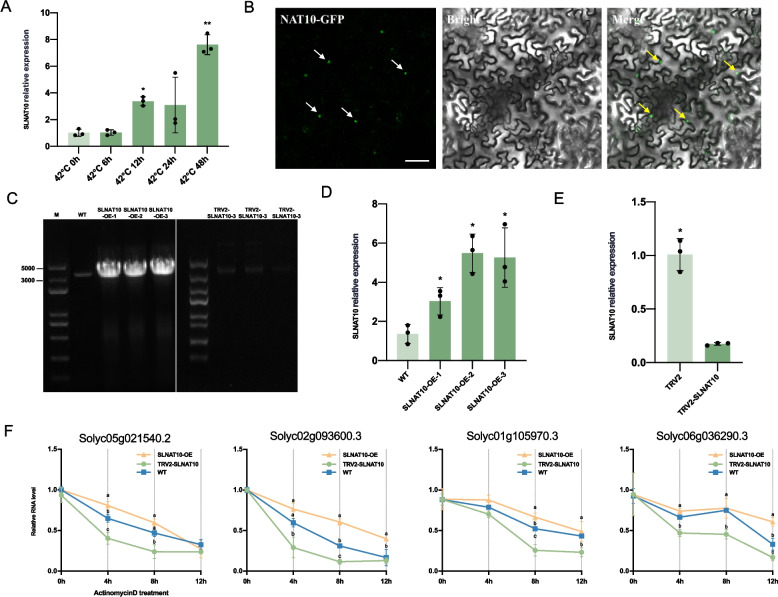
SLNAT10 regulates mRNA stability under heat stress in *S. lycopersicum*. **A** Relative expression levels of SLNAT10 in tomato leaves after heat treatment (42 °C) at 0, 6, 12, 24, and 48 h, showing dynamic transcriptional changes in response to heat stress. The relative expression was quantified by RT-qPCR using actin as the reference gene (ΔCt normalization), and further normalized to the 0 h time point of the same experiment. Statistical analysis was performed using one-way analysis of variance (ANOVA) (* p < 0.05, ** p < 0.01). The data are presented as the means ± standard deviations (SD) (*n* = 3 biological replicates). **B** Confocal microscopy analysis revealed nuclear localization of SLNAT10-GFP in epidermal cells of SLNAT10-OE plants. Scale bar = 20 μm. **C** RT‑PCR validation of SLNAT10 expression. Agarose gel electrophoresis of RT‑PCR confirming successful transcription of the SLNAT10 transgene in SLNAT10‑OE plants and silencing in the TRV2‑SLNAT10 line. **D** RT‑qPCR validation of SLNAT10 relative expression. Relative expression levels of SLNAT10 in the wild type (WT) and three independent SLNAT10-OE lines, with all the transgenic lines exhibiting significantly higher SLNAT10 expression compared to the WT line. Statistical analysis was performed using one-way analysis of variance (ANOVA) (* p < 0.05, ** p < 0.01). The data are presented as the means ± standard deviations (SD) (*n* = 3 biological replicates). **E** Relative expression levels of SLNAT10 in TRV2-SLNAT10-silenced plants, with an empty vector (TRV2) used as the negative control. Statistical analysis was performed using one-way analysis of variance (ANOVA) (* p < 0.05, ** p < 0.01). The data are presented as the means ± standard deviations (SD) (*n* = 3 biological replicates). **F** RT‑qPCR analysis of Solyc05g021540.2, Solyc02g093600.3, Solyc01g105970.3, and Solyc06g036290.3 transcript levels in SLNAT10-OE, WT, and TRV2-SLNAT10 plants after 12 h of heat stress (42 °C), followed by treatment with actinomycin D for 0, 4, 8, and 12 h, demonstrating the effects of SLNAT10 on transcript stability under heat stress conditions. Different letters indicate significant differences between the two groups, as determined by one-way ANOVA (p < 0.05, was to indicate statistical significance). The data are presented as mean ± SD (*n* = 3 biological replicates)

The abundance of the acetyltransferase that is responsible for ac^4^C modification was significantly increased after heat treatment (Fig. 6A). Therefore, we generated three independent transgenic tomato lines overexpressing SLNAT10 (SLNAT10-OE) by genetic transformation. The expression of NAT10-GFP in these lines was visualized using confocal laser scanning microscopy (Fig. 6B), and the overexpression of SLNAT10 was confirmed by both RT-PCR and RT‑qPCR analyses (Fig. 6C–D). In parallel, we employed a TRV-based virus-induced gene silencing (VIGS) system to achieve systemic silencing of SLNAT10, and successful gene knockdown was confirmed by RT‑qPCR (Fig. 6E). Using these genetic materials, we subsequently assessed the transcript stability of selected target genes. After SLNAT10-OE, TRV2-SLNAT10, and WT plants were subjected to 12 h of heat treatment, the relative expression levels of four target genes were measured following actinomycin D infiltration at 4 h, 8 h, and 12 h by RT‑qPCR. The results demonstrated that the transcription of the target genes in the TRV2-SLNAT10 lines decreased rapidly within 12 h of actinomycin D treatment, whereas the transcript levels remained stable in the SLNAT10-OE lines (Fig. 6F). These observations indicate that SLNAT10 positively contributes to the stabilization of specific mRNA transcripts under heat stress conditions.

## Discussion

The ac^4^C modification was first discovered in yeast tRNA through chemical analysis (Rafalski et al. 1979). Decades after its initial identification, researchers characterized NAT10 as the “writer” enzyme that is responsible for ac^4^C modifications and revealed that its activity is driven by ATP hydrolysis (Ito et al. 2014). However, these studies have largely focused on noncoding RNAs, with research on ac^4^C modifications in coding RNAs remaining stagnant for an extended period (Zhou et al. 2013). One major challenge is the low abundance of ac^4^C in vivo compared with m^6^A, making its detection highly challenging (Zhang et al. 2024). In plants, ac^4^C modifications in mRNA were largely unrecognized until 2023. Recent studies have demonstrated the presence of ac^4^C modifications in *A. thaliana* and *O. sativa* mRNA (Li et al. 2023), The conserved genomic distribution of ac^4^C across species, evolutionary conservation of NAT10 sequences, and their shared role in promoting RNA translational efficiency collectively suggest that ac^4^C is a conserved regulatory mechanism in both plants and mammals.

In this study, we systematically profiled ac^4^C modifications in tomato mRNA using acRIP-seq technology. Our results confirmed the presence of ac^4^C modifications in tomato leaf mRNA. In all IP samples, ac^4^C signals were detected, and their genomic distribution was successfully mapped. ac^4^C modifications in tomato mRNA are distributed across all chromosomes, are enriched around initiation codons, and are associated with a conserved CNN sequence motif. Comparative functional enrichment analysis of significantly modified genes in *A. thaliana* and *O. sativa* revealed substantial functional conservation of ac^4^C-targeted genes in plants. These genes are predominantly enriched in pathways related to RNA binding, protein binding, and GTPase activity.

To investigate the role of ac4C modification in plant stress responses, we employed acRIP-seq technology to analyze tomato under heat stress condition. The results indicate that heat stress is associated with increased ac^4^C modification levels in mRNA, with a modest but more apparent enrichment in the 3'UTR, particularly near the stop codon. These findings suggest that ac^4^C modifications might increase translation efficiency and accuracy, thereby facilitating the expression of stress-responsive genes and enabling plants to better cope with high-temperature stress. In mammals, ac^4^C modifications in mRNA have been shown to increase transcript stability and translation efficiency (Arango et al. 2018). Similarly, studies in plants have indicated that ac^4^C-modified transcripts tend to exhibit longer half-lives and higher translation efficiency (Wang et al. 2023). Our experimental findings further substantiate the potential link between ac^4^C modifications and RNA stability under heat stress condition. Genes whose expression increased under heat stress conditions exhibited a corresponding increase in transcript abundance, and these genes were predominantly enriched in pathways associated with environmental stress responses. Consistent with these findings, the silencing of SLNAT10 leads to decreased stability of highly acetylated transcripts under heat stress conditions. Consequently, these transcripts display markedly reduced longevity and are subject to accelerated degradation upon exposure to elevated temperatures, highlighting the essential role of ac^4^C in maintaining mRNA integrity during plant stress responses.

Despite these insights, several unresolved questions remain. Given the critical role of ac^4^C modification in mRNA metabolism, future studies could use high-resolution spatial transcriptomic techniques to systematically investigate the colocalization characteristics of the NAT10 and its target mRNAs at the levels of tissue specificity and cellular heterogeneity, thereby elucidating their spatiotemporal regulatory mechanisms. Furthermore, although NAT10-mediated ac^4^C modification has been well characterized, the “reader” proteins that are responsible for specifically recognizing ac^4^C and determining its biological effects remain unidentified. Future research may integrate RNA‒protein coprecipitation with mass spectrometry-based proteomic analyses to systematically identify candidate proteins. Collectively, advancements in these research directions will increase our understanding of the regulatory networks that are orchestrated by NAT10-mediated RNA modifications in plant development and environmental adaptation.

## Materials and methods

### Plant material and growth conditions

Six-week of *S. lycopersicum* were used in this experiment. The cultivation process involved a consistent photoperiod of 16 h of light and 8 h of darkness, with the temperature maintained at 25 °C and the relative humidity set at 60%.

For heat stress treatment, a subset of plants was exposed to 42 °C for 12 h under continuous light, while control plants remained at 25 °C with identical humidity and photoperiod conditions. Leaf tissues from both HS-treated and control groups were harvested immediately post-treatment for subsequent analyses.

To generate with stable overexpression and transient silencing tomato lines. The SLNAT10 CDS was cloned and inserted into the Gateway-compatible vector Gateway100 under the CaMV 35S promoter, its sequence was verified, and it was introduced into *Agrobacterium tumefaciens* (GV3101) for transformation of tomato cotyledon explants. After cocultivation, explants were selected on MS medium supplemented with kanamycin and cefotaxime, regenerated and rooted on selective medium. T0 plants were subsequently transferred to soil and advanced to T1 by selfing. Transgenic plants were confirmed by PCR targeting the selectable marker, expression levels were validated by RT-qPCR (normalized to actin), and at least three independent homozygous lines with robust SLNAT10 overexpression were selected for downstream analyses. For gene silencing, Agrobacterium-mediated leaf infiltration with the TRV VIGS system (pTRV1 + pTRV2-SLNAT10) was employed.

### Plasmid constructs

To generate 35S:SLNAT10-GFP constructs, the full-length coding sequence of *SLNAT10* was amplified from the cDNA of *S. lycopersicum* and cloned and inserted into the pCAMBIAsuper1300-GFP vector using In-Fusion cloning (ClonExpress II One-Step Cloning Kit, Vazyme).

### RNA extraction and RNA dot blotting

For RNA extraction, 200 mg of treated leaves were snap-frozen in liquid nitrogen, homogenized using a grinder, and then extracted with TRIzol reagent (Vazyme). RNA concentration and purity were assessed using a NanoDrop2000 by the measuring absorbance at 260 nm and 280 nm. Reverse transcription was carried out with TransScript® All-in-One First-Strand cDNA Synthesis SuperMix (Vazyme).

ac^4^C modification of RNA samples was validated using a dot blotting assay. RNAs were heated at 70 °C for 10 min, and 1 µL of each sample or dilution was spotted onto a nitrocellulose membrane. The membrane was air-dried and cross-linked using a UV cross-linker at 120 mJ/cm^2^ for 30 s to crosslink the RNA. The membrane was blocked with TBST containing 5% BSA for 1 h at room temperature, followed by overnight incubation at 4 °C with anti-ac^4^C antibody (1:500 dilution) (Abcam). After three washes with 1 × TBST, the membrane was incubated for 1 h with an HRP-conjugated anti-rabbit secondary antibody (1:5000 dilution). Chemiluminescent detection was performed using Immobilon Western Chemiluminescent HRP Substrate, and fluorescence signals were visualized by a GelCap image system (Bio-Rad).

### Library construction for RNA-Seq and acRIP-Seq

RNA-seq and acRIP-Seq were performed by Cloudseq Biotech, Inc. (Shanghai, China). Briefly, poly(A) + RNA was first purified from total RNA using the GenElute mRNA Miniprep Kit (Sigma**–**Aldrich), followed by additional depletion of residual rRNA using the Ribo-Zero kit (Illumina) prior to library preparation. acRIP was performed with a GenSeq ac^4^C RIP Kit according to the manufacturer’s instructions. Both acRIP and input samples were used for library generation with a NEBNext Ultra II Directional RNA Library Prep Kit (New England Biolabs). The library was validated using an Agilent 2100 bioanalyzer and sequenced on a HiSeq 4000 instrument (Illumina).

### Analysis of sequencing data

The sequencing data were processed for quality control (QC) using fastp software (version 0.23.0). During library preparation, each molecular fragment was randomly tagged with a unique identifier (UID) sequence. After the raw data were subjected to QC, UID processing software (kcUID) was used to cluster similar reads under the same UID, enabling error correction and the removal of PCR duplicates. This ensured more accurate molecular sequence information and expression quantification. The high-quality clean reads were first aligned to the ribosomal RNA (rRNA) database of the target species using Bowtie2, and rRNA reads were subsequently removed. The remaining reads were subsequently mapped to the reference genome (https://solgenomics.net/ftp/tomato_genome/annotation/ITAG3.2_release/) using HISAT2. Peak calling was performed with the exomePeak tool to identify enrichment peaks. The genomic distribution of peaks was analyzed to determine their localization in coding sequences (CDS), intergenic regions, or intronic regions, along with functional annotation of the associated genes. Homer was used to extract sequence regions corresponding to peaks, scan for shared motifs, identify target binding motifs, and construct motif logos. Differential peak analysis between comparison groups was carried out using DESeq2 software, with thresholds set at an input number ≥ 2 and p value < 0.05. Gene expression levels were quantified as RPKM. To annotate gene functions and pathways, GO and KEGG enrichment analyses were conducted. Finally, data visualization and presentation were completed using Prism 8 software.

### acRIP-RT-qPCR

For acRIP, 500 μg of total RNA was incubated with anti-ac^4^C antibodies (Abcam) or IgG antibodies (Beyotime) in 500 μL of IP buffer (150 mM NaCl, 0.1% NP-40, 10 mM Tris–HCl, pH 7.4) for 4 h at 4 °C. The mixtures were incubated with 30 μL of anti-rabbit antibodies conjugated with magnetic beads (NEB) for 2 h at 4 °C and then were washed six times with 1 ml of IP buffer. RNA was extracted using TRIzol reagent and quantified by RT‑qPCR.

RT-qPCR was performed using 2 × ChamQUniversal SYBR qPCR Master Mix (Vazyme), on an Applied Biosystems 7500 Fast Real-Time PCR System. The relative expression levels of target genes were calculated using the 2^−ΔΔCt^ method, while the modification levels of the transcripts were assessed using the % input method. The % input was calculated using the formula:

$$\% Input=\frac{{2}^{({Ct}_{Input}-{Ct}_{RIP})}}{dilution factor}\times 100$$

Ct_Input_ and Ct_RIP_ represent the Ct values of the input and IP or IgG samples, respectively, and the dilution factor is 10. All the reactions were performed in triplicate, and the results are expressed as the mean ± standard deviation (SD) from three independent biological replicates. All the primers used for RT‑qPCR are listed in Supplementary material 2.

### Analysis of mRNA decay

*S. lycopersicum* leaves were treated with 30 µM actinomycin D (Sigma‒Aldrich). Total RNA was extracted from 200 mg of leaf tissue at 0-, 4-, 8-, and 12-h post-treatment using TRIzol. cDNA was synthesized via reverse transcription and used to assess the stability of the candidate genes. All the primers used for RT‑qPCR are listed in Supplementary material 2.

## Supplementary Information

Supplementary Material 1. Figure S1. Phylogenetic and sequence conservation analysis of RNA cytidine acetyltransferases. Figure S2. The full‑length membrane image from Figure 1C. Figure S3. Total RNA integrity assessment by agarose gel electrophoresis. Figure S4. Validation of two hyper-down genes by RT‑qPCR and acRIP‑RT‑qPCR.

Supplementary Material 2. Table S1. List of primers that were used in this study. Table S2. Quality control of acRIP-seq. Table S3. Hypomethylated ac^4^C peaks identified in *S. lycopersicum*.

## Data Availability

All sequencing raw data generated in this study have been deposited in the Genome Sequence Archive (GSA) at the China National Center for Bioinformation (CNCB). The accession number for the acRIP-seq data is CRA031153, and the accession number for the RNA-seq data is CRA031249. These datasets are publicly accessible at https://ngdc.cncb.ac.cn/gsa.

## Abbreviations

ac^4^C: Cytosine N4 Acetylation
acRIP-seq: Acetylated RNA immunoprecipitation sequencing
HS: Heat stress
RPKM: Reads per kilobase of transcript
GO: Gene Ontology
KEGG: Kyoto Encyclopedia of Genes and Genomes
DEGs: Differentially expressed genes
ANOVA: Analysis of variance
WT: Wild type
RT‑qPCR: Quantitative real-time polymerase chain reaction
OE: Overexpression
