# Supplementary material for: Epitranscriptomic profiling of cytosine N4 acetylation (ac4C) in Solanum lycopersicum and dynamic changes under heat stress condition
Source: Mol Hortic. 2026 May 6;6:37. doi: 10.1186/s43897-025-00214-7 (PMC13147876; doi:10.1186/s43897-025-00214-7)
Supplement: Supplementary file 1 — Supplementary Material 1. Figure S1. Phylogenetic and sequence conservation analysis of RNA cytidine acetyltransferases. Figure S2. The full‑length membrane image from Figure 1C. Figure S3. Total RNA integrity assessment by agarose gel electrophoresis. Figure S4. Validation of two hyper-down genes by RT‑qPCR and acRIP‑RT‑qPCR. [file 43897_2025_214_MOESM1_ESM.docx]

Figure S1. Phylogenetic and sequence conservation analysis of RNA cytidine acetyltransferases.

A. Protein sequence alignment of SLNAT10 with yeast Kre33, human NAT10, and Arabidopsis AT3G57940 and AT1G10490. Dark blue regions indicate highly conserved amino acid residues.

B. Phylogenetic tree of the RNA cytidine acetyltransferase protein sequences from different species. *Nicotiana benthamiana* RNA cytidine acetyltransferase (Niben101Scf06641g01005.1), *Nicotiana sylvestris* RNA cytidine acetyltransferase (LOC104248816), *Nicotiana attenuata* RNA cytidine acetyltransferase (LOC109225321), *Capsicum annuum* RNA cytidine acetyltransferase (LOC107850717), *Anisodus acutangulus* RNA cytidine acetyltransferase (A0A9Q1LT72), *Solanum verrucosum* RNA cytidine acetyltransferase (LOC125812644), *Sorghum bicolor* RNA cytidine acetyltransferase (LOC8083812), *Zea mays* RNA cytidine acetyltransferase(LOC109941429), *Malus domestica* RNA cytidine acetyltransferase (LOC103444309), *Prunus persica* RNA cytidine acetyltransferase (LOC18773206), *Solanum lycopersicum* RNA cytidine acetyltransferase (Solyc04g051670.2), *Abrus precatorius* RNA cytidine acetyltransferase (LOC113872446), *Capsella rubella* RNA cytidine acetyltransferase (LOC17900849), *Arabidopsis thaliana* RNA cytidine acetyltransferase (A0A8T2GCN4), *Panicum hallii* RNA cytidine acetyltransferase (LOC112886901), *Arachis hypogaea* RNA cytidine acetyltransferase (LOC112733215).

Figure S2. The full‑length membrane image from Figure 1C. Western blotting results showing that transient overexpression of SLNAT10 fused to GFP yields a specific immunoreactive band with an anti‑GFP antibody, whereas the WT sample shows no band at approximately 145 kDa.

Figure S3. Total RNA integrity assessment by agarose gel electrophoresis. Lanes 1–3 contain total RNA extracted from tomato leaves grown under 25℃ (control), and lanes 4–6 contain total RNA from tomato leaves under heat stress condition at 42℃.

Figure S4. Validation of two hyper-down genes by RT‑qPCR and acRIP‑RT‑qPCR.

A-B. RT‑qPCR results show significant reductions of the transcription levels of the two candidate genes under heat stress condition. The data represent the mean ± SD of three biological replicates. Statistical significance was determined using one-way ANOVA (*p < 0.05, **p < 0.01).

C-D. acRIP-RT-qPCR results show a corresponding decrease in ac⁴C modifications for the same genes. The data represent the mean ± SD of three biological replicates. Statistical significance was determined using one-way ANOVA (*p < 0.05, **p < 0.01)
